# p38α regulates actin cytoskeleton and cytokinesis in hepatocytes during development and aging

**DOI:** 10.1371/journal.pone.0171738

**Published:** 2017-02-06

**Authors:** Ana M. Tormos, Sergio Rius-Pérez, María Jorques, Patricia Rada, Lorena Ramirez, Ángela M. Valverde, Ángel R. Nebreda, Juan Sastre, Raquel Taléns-Visconti

**Affiliations:** 1 Department of Physiology, University of Valencia. Burjassot, Valencia, Spain; 2 Instituto de Investigaciones Biomédicas Alberto Sols (Centro Mixto CSIC-UAM), Arturo Duperier 4, Madrid, Spain; 3 Centro de Investigación Biomédica en Red de Diabetes y Enfermedades Metabólicas Asociadas (CIBERdem), ISCIII, Madrid, Spain; 4 Institute for Research in Biomedicine (IRB Barcelona), Barcelona Institute of Science and Technology, Barcelona, Spain; 5 Institució Catalana de Recerca i Estudis Avançats (ICREA), Barcelona, Spain; 6 Department of Pharmacy and Pharmaceutical Technology and Parasitology, University of Valencia. Burjassot, Valencia, Spain; University of Navarra School of Medicine and Center for Applied Medical Research (CIMA), SPAIN

## Abstract

**Background:**

Hepatocyte poliploidization is an age-dependent process, being cytokinesis failure the main mechanism of polyploid hepatocyte formation. Our aim was to study the role of p38α MAPK in the regulation of actin cytoskeleton and cytokinesis in hepatocytes during development and aging.

**Methods:**

Wild type and p38α liver-specific knock out mice at different ages (after weaning, adults and old) were used.

**Results:**

We show that p38α MAPK deficiency induces actin disassembly upon aging and also cytokinesis failure leading to enhanced binucleation. Although the steady state levels of cyclin D1 in wild type and p38α knock out old livers remained unaffected, cyclin B1- a marker for G2/M transition- was significantly overexpressed in p38α knock out mice. Our findings suggest that hepatocytes do enter into S phase but they do not complete cell division upon p38α deficiency leading to cytokinesis failure and binucleation. Moreover, old liver-specific p38α MAPK knock out mice exhibited reduced F-actin polymerization and a dramatic loss of actin cytoskeleton. This was associated with abnormal hyperactivation of RhoA and Cdc42 GTPases. Long-term p38α deficiency drives to inactivation of HSP27, which seems to account for the impairment in actin cytoskeleton as Hsp27-silencing decreased the number and length of actin filaments in isolated hepatocytes.

**Conclusions:**

p38α MAPK is essential for actin dynamics with age in hepatocytes.

## Introduction

Polyploid cells contain more than two complete sets of chromosomes and they are very common in liver tissue [1–3]. Hepatocyte poliploidization is considered an age dependent-process that occurs mainly during liver development and postnatal maturation. However, poliploidization changes can take place also in adult liver as a result of increasing age or cellular stresses such as surgical resection, toxic exposure or viral infections [3]. In any case, the poliploidization process generates tetraploid or octoploid hepatocytes with one (mononucleated hepatocytes) or two nuclei (binucleated hepatocytes) [2, 3]. Although several mechanisms of polyploid cell formation have been reported in mammals, including endoreduplication, endomitosis and mitotic slippage, incomplete cytokinesis is the main mechanism of polyploid hepatocyte formation in the liver [1–4]. Physiological failure of cytokinesis in the liver occurs after the end of the suckling (weaning), associated with insulin signaling *via* the phosphatidylinositide 3 kinase—protein kinase B (PI3K-AKT) pathway [5] and increases progressively with age [6, 7]. Therefore, most hepatocytes are diploid in young individuals, while more than half are polyploid in adults [8].

The cleavage of the mother cell into two daughter cells during cytokinesis, the last step of cell division, implies re-organization of the actin cytoskeleton to assembly the myosin and actin contractile ring that allows the formation of the cleavage furrow [9–14]. Formation of the cleavage furrow is a critical structure for successful cytokinesis execution and must be regulated properly to ensure that chromosomes and organelles are distributed equally to each daughter cell [15]. Filamentous actin (F-actin) together with additional structural and regulatory proteins plays an important role in the cleavage furrow formation. Localized activation of the small GTPases Ras homolog (Rho) family, including RhoA, Cdc42 and Rac1 are essential for furrow formation in animal cells promoting actin polymerization and stimulating myosin activity [16–19]. Insufficient activation of this pathway could perturb proper initiation of cytokinesis and could induce cytokinesis failure. On the other hand, the role of cofilin in actin depolymerization to maintain actin dynamics is essential for cytokinesis completion [18, 19].

Cytokinesis failure results in the formation of genetically unstable polyploid cells so understanding the mechanisms of cytokinesis is a central problem in cell biology with potential relevance in tumorigenesis [12]. Paradoxically, during postnatal development of the liver, polyploid hepatocytes may be generated physiologically as a result of failures either in the cytoskeleton reorganization or in activation of molecular signals essential for hepatocyte cleavage [3].

The binucleation ratio of the liver seems to be directly connected with the proliferative capacity of hepatocytes. During postnatal liver growth, when poliploidization is established, hepatocytes exhibit lower ability to proliferate. This occurs because many hepatocytes manage to finish karyokinesis, but most of them fail to complete cytokinesis thus generating binuclear cells [2, 3]. Interestingly, hepatocytes are able to change from polyploid to diploid, and from binucleated to mononucleated during organism’s life by a phenomenon called somatic ‘reductive mitoses’, thanks to multipolar mitotic spindles [20]. In fact, ploidy reversal is a useful tool to enhance hepatocyte proliferation, which is especially beneficial after liver hepatectomy when a rapid liver tissue growth is required [3] [21].

We have already observed that binucleation reversal after biliary cirrhosis was reduced upon p38α deficiency showing the key role of p38α in hepatocyte proliferation [22]. The p38 MAPK family consists of 4 members: p38α, p38β, p38γ (SAPK (stress-activated protein kinase) 3, and p38δ (SAPK4) [23], being p38α ubiquitously expressed at high levels in most cell types [24]. p38 MAPKs are activated by environmental and genotoxic stress and play key roles in the control of cell proliferation, differentiation and survival, as well as in the regulation of the inflammatory response [25],[22]. In fact, p38 MAPKs participate in inflammatory diseases such as rheumatoid arthritis [26], Crohn’s disease [27], asthma [28] and chronic obstructive pulmonary disease [29], as well as in other pathological conditions like cardiovascular diseases [30, 31], cancer [32, 33], or pain [34, 35]. Accordingly, p38 MAPK inhibitors have demonstrated adequate properties to treat inflammatory diseases and other conditions. Several p38 MAPK inhibitors, most of which have reached phase 2 clinical studies, have been developed [36, 37] [38–42]. However, many of them have been discontinued due to adverse effects such as gastrointestinal disorders, liver anomalies and ALT elevations, among others [35] [40, 41].

Regarding the regulation of hepatocyte proliferation by p38α, it is known that p38α regulates the G_1_/S and G_2_/M cell-cycle checkpoints prior to DNA synthesis and cell division, respectively [43–45]. So far increased proliferation and impaired differentiation have been considered hallmarks of p38α-deficient cells [46]. Thus, mice with liver-specific deletion of p38α exhibited enhanced hepatocyte proliferation after partial hepatectomy [47] and developed more liver tumors with increased number of proliferative tumor cells [48]. Accordingly, activation of p38 MAPK resulted in hepatocyte growth arrest and inhibition of DNA synthesis in cultured fetal rat hepatocytes [43]. In addition, inhibition of p38 MAPK *in vivo* is sufficient to trigger a marked increase in the number of proliferating hepatocytes [43]. Nevertheless, paradoxically we recently found that liver-specific p38α deficiency lowered hepatocyte proliferation and enhanced hepatocyte binucleation in biliary cirrhosis [22], which suggested that p38α might influence the last step of mitosis. Therefore, in the present manuscript we have explored in more depth the role of p38α in the regulation of cytokinesis progression and actin cytoskeleton in hepatocytes during development and aging.

## Materials and methods

### Animals

p38α was specifically down-regulated in the hepatocytes by using mice carrying *p38α* floxed alleles1 and the Afp-Cre transgene that expresses Cre under the control of the α-fetoprotein promoter, which is active during embryonic hepatic development. The liver-specific p38α knock out (KO) mice were kept in a C57BL/6 genetic background [49]. We performed experiments with wild type and p38α knock out mice at three different ages: after weaning (4 weeks-old), adults (10–12 weeks-old) and old (18–24 months-old). Four to six animals were used for each experimental group.

All mice were cared for in accordance with the criteria outlined in the Guide for the Care and Use of Laboratory Animals (NIH publication 86–23 revised 1985) and they were cared under controlled conditions of temperature (23±1°C), relative humidity (50–60%) and light/dark cycles (12h/12h) with food and water *ad libitum*. Mice were anesthetized with isoflurane 3–5% and once they were unconscious they were exsanguinated. Death was confirmed by cervical dislocation. The study was approved by the Ethics Committee of Animal Experimentation and Welfare of the University of Valencia (Valencia, Spain).

### Primary hepatocyte cell culture

Primary mouse hepatocytes were isolated from non-fasting male C57BL/6 mice (3–4 months) by a two-step collagenase perfusion as previously described [50]. Briefly, cells were seeded on collagen-coated 12-well plate (Corning, Inc.) and cultured at density of 350,000 cells/well in 1 ml medium containing Dulbecco's modified Eagle's medium and Ham's F-12medium (1:1) supplemented with 10% FBS, 2 mM glutamine, 100 units/ml penicillin, 100 μg/ml streptomycin, and 1 mM sodium pyruvate (attachment medium) and maintained under a humidity conditions in 95% air and 5% CO_2_ at 37°C for 24 h before siRNA transfection. For cell staining experiments, collaged-coated coverslips were used.

### Genes knockdown by siRNA

Cells were transfected with 25 nM siRNAs or with a scrambled siRNA, used as control, following DharmaFECT General Transfection Protocol (Dharmacon) to knock down mouse p38α and Hsp27 expression. Cells were used 48 h later for experiments. *DharmaFECT siRNA Transfection Reagent*, p38α siRNA (SMARTpool: ON-TARGETplus Mapk14 siRNA L-040125-00-0005 5 nmol), Hsp27 siRNA (SMARTpool: ON-TARGETplus Mouse Hspb1 siRNA L-045651-00-0005 5 nmol) and scramble siRNA were obtained from Dharmacon.

### Western blotting

For total liver homogenates, protein was extracted with Heidolph (RZR 2021) homogenizer in Hepes lysis buffer pH 7.4 with 1mM DTT, 1mM sodium ortovanadate, 50mM sodium fluoride, 30mM sodium pyrophosphate, 1% Igepal, 10% glycerol and protease inhibitor cocktail (Sigma Aldrich). To obtain total cell lysates, attached cells were scraped off and incubated for 10 min on ice with lysis buffer. Homogenates and cell lysates were centrifuged during 15 min at 15000 rpm, 4°C. In case of nuclei isolation, a slight modification of the nuclei isolation method described by [51] was used. Chemiluminescence was detected with a charge-coupled device camera (Biorad ChemiDoc XRS+ Molecular Imager and LAS-3000, Fujifilm) using the ECL system (Luminata Classico, Millipore). Antibodies used were as follows: AKT (Genscript A00301); α tubulin (Sigma Aldrich T6074); cyclin B1 (sc-245), cyclin D1 (sc-20044), GSK3β (sc-9166), HSP27 (sc-59562), MK2 (sc-7871), p21 (sc-6246), p38 alpha (sc-535), p-GSK3β (BS4084) from Santa Cruz Biotechnology; tata binding protein (abcam, 818); MNK1 (Novus biological, H00008569-M14); p-AKT (Ser473) (4058), β catenin (9562), p-cofilin (Ser 3) (3313), cofilin (5175), p-HSP27 (Ser82)(2401), p-H3 (Ser10) (3377), H3 (4499), p-MKK3/6 (Ser189/207) (9231), p-MKK4 (Ser257/Thr261) (9156), p-MK2 (Thr334) (3007), p-MK2 (Thr222) (3316), p-p38 (Thr 180/Tyr188) (4511XP), p-MNK1(Thr197/202) (2111), p27 (2552), from Cell Signaling Technology. Secondary antibodies were from Jackson Inmunoresearch: Donkey anti rabbit (711-035-152), donkey anti mouse (715-035-151) and donkey anti goat (715-035-151).

### F-actin polymerization assay

Frozen livers were used for this assay, as no differences were found between fresh and frozen livers. This technique was performed as indicated by manufacturer (BK003 Cytoskeleton). Briefly, after stabilizing actin using the buffer provided by the manufacturer, the liver tissue was homogenized and the proteins extracted as described above. The lysate was centrifuged at 350 g for 5 minutes at room temperature. Then, the supernatant was centrifuged at 100,000 g, 1 h at 37°C to obtain F-actin/G-actin fractions which were quantified by Western blotting.

### GTPase activity measurement for RhoA, Cdc42, Rac1

The measurement was performed as described by the manufacturer (BK036, BK034, and BK035 respectively). Frozen livers were homogenized and total lysates were centrifuged at 10000g, 1min, 4°C, as preclearing. Once bead titration was accomplished, immunoprecipitation was performed with 15uL of beads and 1mg/mL of liver lysate in the case of Rhotekin RBD beads and 10uL of beads and 0.5mg/mL of liver lysate in the case of PAK-PBD beads. All of them required 1mL final volume.

### Immunohistochemistry and cell staining

Formalin-fixed, paraffin embedded sections of liver tissue were deparaffinised using Histo-Clear® (National Diagnostics). As an alternative for xylene and antigen retrieval, tissue sections were autoclaved in citrate buffer pH 6 for β-catenin staining or digested with proteinase K in TE buffer at 37°C, in the case of filamentous actin (F-actin). Slides were blocked with 5% BSA in PBS. Antibodies used in immunofluorescence were β-catenin (Cell Signaling Technology, 9562) and F-actin (Acris antibodys, SM1349P). Nuclei were stained with DAPI (Life Technologies, D1306). For binucleation rate (percentage of binucleated cells/ total number of cells) and number of nuclei per field, 50–60 slides from all different animals were blindly scored.

For *in vitro* assays, primary hepatocytes seeded on collagen-coated glass coverslips (10 mm) in 12-well plates were fixed with 4% paraformaldehyde for 6 min, washed with PBS and permeabilised with 0,1% Triton X-100 during 5 min. Actin was detected by incubating cells for 1 h with Texas Red®-X phalloidin (ThermoFisher) and nuclei were stained with DAPI. An OLYMPUS FV1000MPE confocal microscope was used for image acquisition.

### TUNEL assay

Formalin-fixed, paraffin embedded sections were deparaffinised using xylene and then, antigen retrieval was performed by citrate buffer (37°C, 15 minutes). Nucleotide labelling and detection were performed as described following manufacturer’s instructions (In Situ Cell Death Detection Kit 11 684 817 910, Roche).

### Statistical analysis

Results are given as mean ± standard deviation (s.d.). Significant differences were assessed by one-way analysis of variance (ANOVA) followed by a Tukey’s *post-hoc* test. Differences were considered statistically significant at p<0.05.

## Results

### Binucleation rises in hepatocytes from liver-specific p38α knock out mice

**Fig 1:**
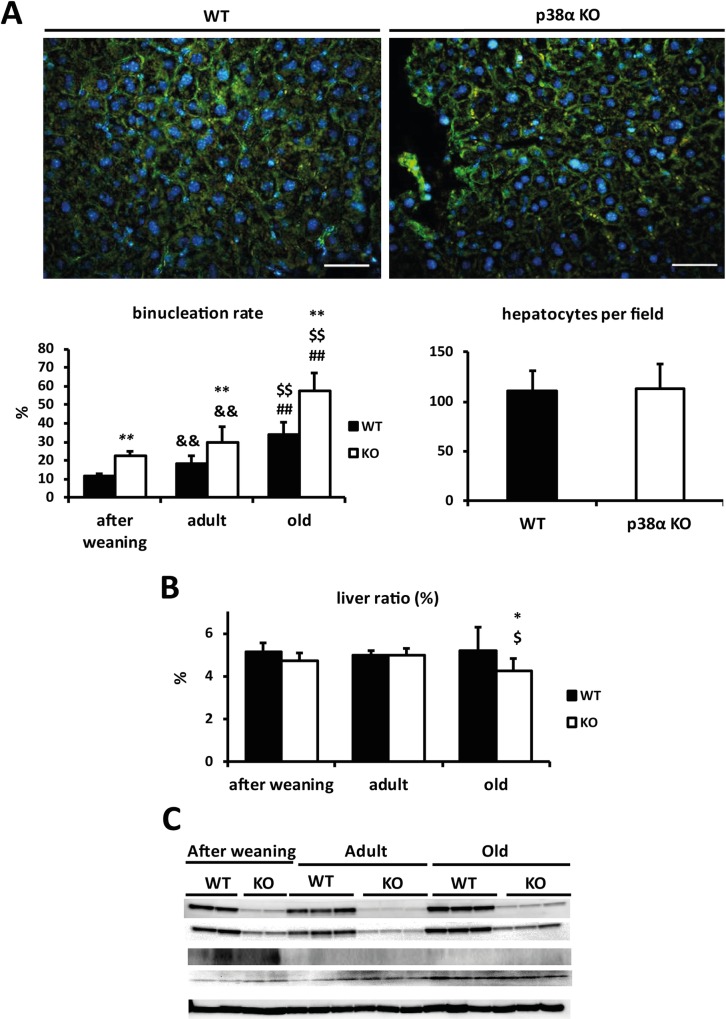
p38α deficiency induces hepatocyte binucleation. **a.** Representative image of β-catenin (green) and DAPI (blue) immunohistochemistry in old wild type and p38α knock out liver mice (Scale bars = 50 μm). Quantification of the binucleation rate (binucleated hepatocytes /total hepatocytes) with age. Number of hepatocytes per field in old wild type and p38α knock out mice as an indirect estimation of hepatocyte size in these animals. **b.** Liver mass ratio with age expressed as the ratio: liver weight/body weight. **c.** Wild type and p38α knock out livers were Western blotted for p-p38 (Thr180/Tyr182), p38α, p-H3 (Ser 10) and H3. α-tubulin was used as a loading control. Data are shown as mean ± SD. *P < 0.05, **P < 0.01 WT *versus* KO; &&P < 0.01 adult *versus* after weaning; $P < 0.05, $ $P < 0.01 old *versus* adult; ##P < 0.01 old *versus* after weaning.

In order to explore the role of p38α in the regulation of cytokinesis in hepatocytes during development and aging, we have determined the binucleation rate by immunohistochemistry in the three groups of mice analysed after weaning, in adult, and in old stages. We have found that p38α deficiency enhanced hepatocyte binucleation at all stages when compared with their corresponding wild type counterparts. The highest binucleation rate (>50%) was found in old p38α knock out mice. Strikingly, the binucleation rate rose with age in both wild type and p38α knock out mice (Fig 1A).

In addition, the liver mass ratio (liver weight/ mouse weight x 100) was determined at different ages of wild type and liver-specific p38α knock out mice, and we found that p38α deficiency reduced liver mass in old mice (Fig 1B). Indeed, although no differences were detected liver mass between after weaning and adult mice, there was a significant decrease in the liver mass of old p38α knock out mice compared with old wild type mice.

To have an estimation of hepatocyte size, the number of hepatocytes per field was determined and it was not affected by p38α deficiency. Therefore, a decline in hepatocyte size should not account for the reduction in liver mass observed in old p38α knock-out animals (Fig 1A).

### Hepatocytes from p38α knock out mice actively enter into mitosis but fail to complete cytokinesis

**Fig 2:**
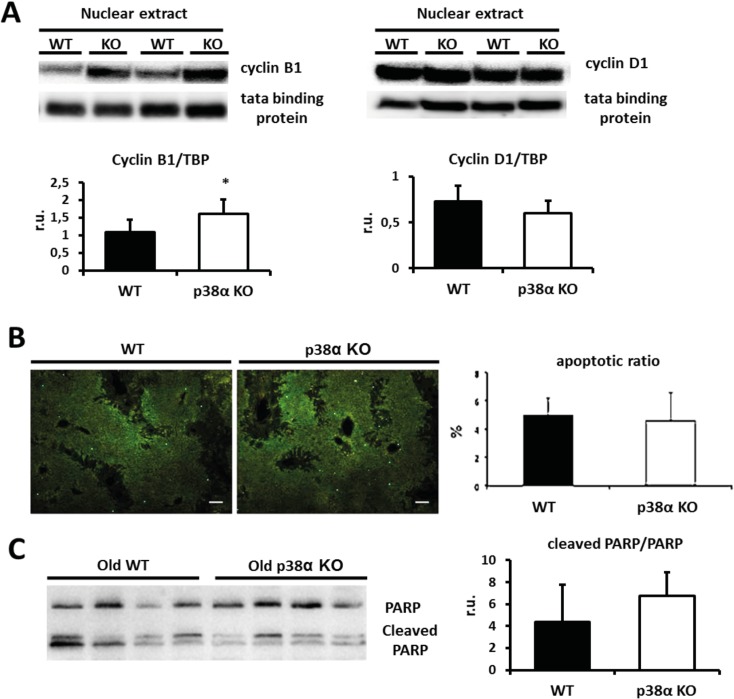
p38α deficiency impairs hepatocyte cell cycle progression and does not activate pro-apoptotic pathways in old liver. **a.** Nuclear fractions from old wild type and p38α knock out liver were Western blotted for cyclin B1 and cyclin D1. Tata binding protein was used as a loading control and densitometric quantification of cyclin D1/TBP and cyclin B1/TBP was performed. **b**. Apoptosis in wild type and p38α knock out old liver sections was measured by the number of positive nuclei using TUNEL (Scale bars = 100 μm). Apoptotic ratio was calculated as: TUNEL positive nuclei/total nuclei. **c.** Old wild type and p38α knock out livers were Western blotted for PARP and cleaved PARP and densitometric quantification of PARP cleavage (cleaved PARP/PARP) was performed. Data are shown as mean and SD. *P < 0.05 WT *versus* KO.

In order to assess whether impaired hepatocyte proliferation due to cell cycle blockade could explain the reduced liver mass upon p38α deficiency and aging, phosphorylated histone 3 (p-H3), cyclin B1 and cyclin D1 were measured in the liver as indexes of proliferative status and potential mitotic delay or blockade. The proliferative rate was markedly increased in livers from both groups only after weaning based on p-H3 levels (Fig 1C). In addition, no significant differences were found in p38α phosphorylation levels upon aging. We decided to focus on old animals because they exhibited major differences in binucleation rates and liver mass. Although the steady state levels of cyclin D1 in wild type and p38α knock out old livers were similar, cyclin B1 was significantly overexpressed in p38α knock out mice (Fig 2A).

On the other hand, the reduced liver mass upon p38α deficiency and aging should not be ascribed to apoptosis, which was not increased upon p38 deficiency. Indeed, cleaved PARP and TUNEL positive hepatocytes were not increased in liver from old p38α knock out mice (Fig 2B and 2C).

### p38α is essential for actin polymerization in hepatocytes upon aging

**Fig 3:**
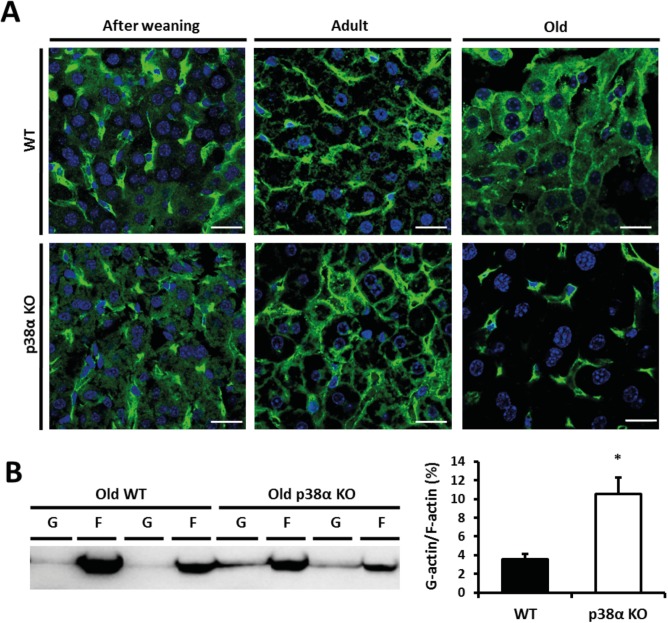
p38α deficiency affects actin polymerization upon aging. **a.** Representative image of F-actin (green) and DAPI (blue) immunohistochemistry in after weaning, adult and old wild type and p38α knock out livers (Scale bars = 10 μm). **b.** F-actin and G-actin immunoblots obtained by ultracentrifugation in old wild type and p38α knock out livers. Densitometric quantification of the G-actin/F-actin ratio was performed. Data are shown as mean and SD. *P < 0.05 WT *versus* KO.

To investigate the possible mechanism underlying the cytokinesis impairment that occurs upon p38α deficiency in the liver, we studied the actin cytoskeleton by immunohistochemistry in livers from wild type and liver-specific p38α knock out mice at all ages. Although after weaning no differences were found, some abnormalities in the cytosolic distribution of actin filaments in adult mice upon p38α deficiency were observed (Fig 3A). A severe impairment in the F-actin filamentous structure was found in the liver from old p38α knock out mice (Fig 3A), as evidenced by dramatic loss of actin cytoskeleton. Furthermore, F-actin polymerization was assessed by separation of F-actin and G-actin fractions, and F-actin was markedly decreased in old p38α knock out mice leading to an increase in G-actin/F-actin ratio in these old mice (Fig 3B).

### Long-term p38α deficiency triggers hyper-activation of RhoA and Cdc42 GTPases

**Fig 4:**
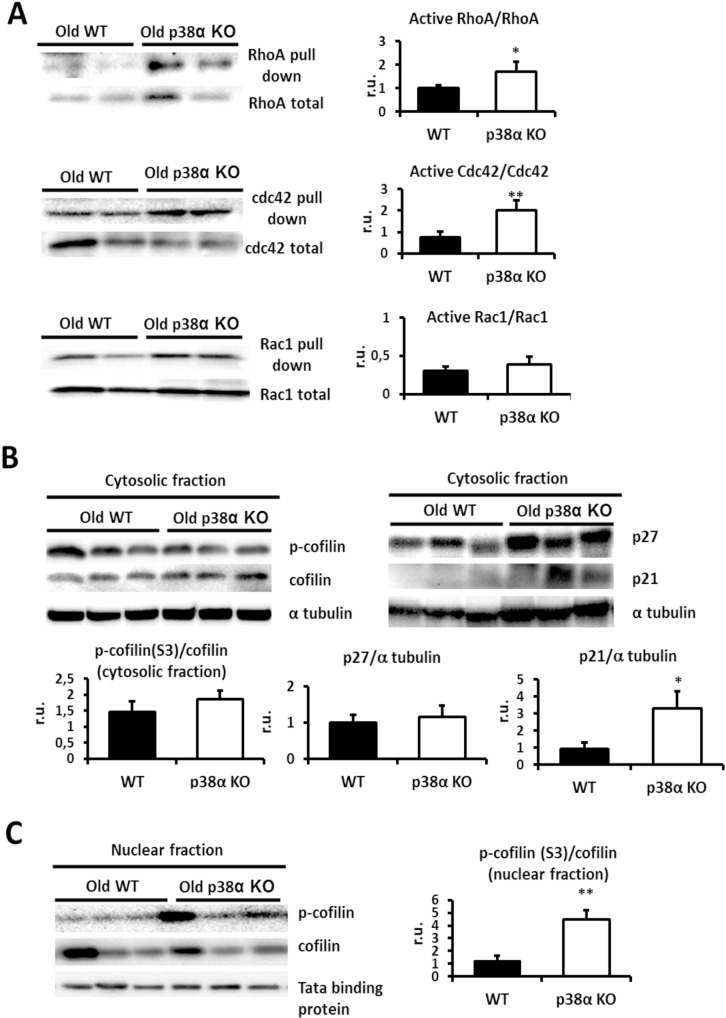
p38α deficiency modulates Rho GTPases. **a.** Old wild type and p38α knock out livers were Western blotted for activated and total levels of RhoA, for activated and for total levels of Cdc42 and for activated and total levels of Rac1. Densitometric quantification of active RhoA/RhoA, active Cdc42/Cdc42, active Rac1/Rac1 was measured. **b.** Old wild type and p38α knock out liver cytosolic fractions were Western blotted for phosphorylated cofilin and total levels of cofilin, p27 and p21. α-tubulin was used as a loading control. Densitometric analysis of p-cofilin(S3)/cofilin, p27/α-tubulin and p21/α-tubulin was done. **c.** Old wild type and p38α knock out liver nuclear fractions were Western blotted for phosphorylated and total levels of cofilin and the densitometric quantification of p-cofilin(S3)/cofilin was performed. Tata binding protein was used as a loading control. Data are shown as mean ± SD. *P < 0.05 WT *versus* KO. **P < 0.01 WT *versus* KO.

The Ras homolog (Rho) family plays a central role in organizing the actin cytoskeleton and in the regulation of cytokinesis [16, 17]. Therefore the GTPase activity of the three major components of Rho GTPases—the Ras homolog A (RhoA), Ras-related C3 botulinum toxin substrate 1 (Rac1), and Cdc42- were measured in the liver of old mice both wild type and p38α knock out. Surprisingly, the activities of RhoA and Cdc42 GTPases increased in old p38α knock out mice in comparison with old wild type mice. No significant changes were found in Rac1 activity (Fig 4A).

### Long-term p38α deficiency increases p21 levels and activates nuclear cofilin

RhoA activity may be inhibited by p27 [52], and additionally the RhoA downstream pathway may be blocked by p21 or cofilin [53, 54]. Hence, p27 and p21 levels as well as the activation of cofilin by phosphorylation were assessed. Nuclear levels of phosphorylated cofilin, but not cytosolic levels, increased upon p38α deficiency in old mice (Fig 4B and 4C). Cytosolic p27 levels did not change significantly, whereas p21 levels increased in the liver of p38α knock out mice (Fig 4B).

### p38α drives activation of MNK1 in old mice

**Fig 5:**
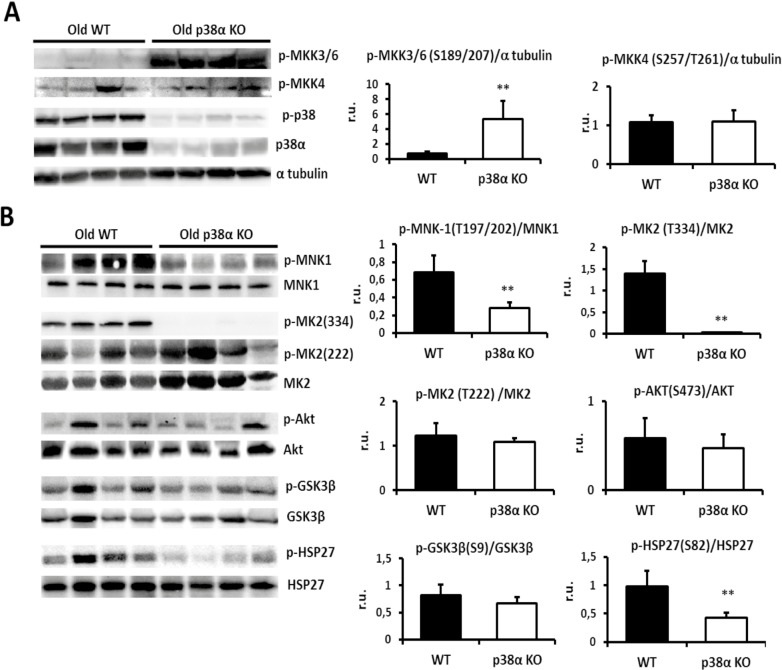
p38α-mediated phosphorylation pathways. **a.** Old wild type and p38α knock out livers were Western blotted for phosphorylated MKK3/6 and MKK4, for phosphorylated p38 and total levels of p38α. Densitometric quantification of p-MKK3/6 (S189/207)/α-tubulin, p-MKK4 (S257/T261)/a tubulin were done. **b.** Old wild type and p38α knock out livers were Western blotted for MNK1 and phosphorylated MNK1 on Thr197/202, for MK2 and phosphorylated MK2 on Thr334 and Thr222, for AKT and phosphorylated AKT on Ser473, for GSK3β and phosphorylated GSK3β on serine 9 and for HSP27 and phosphorylated HSP27 on Ser 82. α-tubulin was used as a loading control. Densitometric quantification of p-MNK-1(T197/202)/MNK1, p-MK2 (T334)/MK2, p-MK2 (T222) /MK2, p-AKT(S473)/AKT, p-GSK3β(S9)/GSK3β and p-HSP27(S82)/HSP27 were determined. Data are shown as mean ± SD. **P < 0.01 WT *versus* KO.

MAPK-interacting Ser/Thr kinase 1 (MNK1) and MAPK-activated protein kinase 2 (MK2)-are major downstream targets of the p38α pathway that have been implicated in the regulation of cytokinesis and actin dynamics [55–57]. Hence their activation was studied in the liver of old wild type and p38α knock out mice as the major changes in cytokinesis and actin cytoskeleton were found in these mice. MNK1 is activated by phosphorylation and is required for abscission of the intercellular bridge at the end of cytokinesis [55]. MNK1 phosphorylation was markedly diminished by p38α deficiency in the liver of old mice (Fig 5B). Hence, MNK1 down-regulation may be involved in the cytokinesis failure that occurs in the liver of these mice.

Strikingly, MK2 phosphorylation on threonine 334 was completely abrogated in the liver from p38α knock out mice, but its phosphorylation on threonine 222 was not affected. However, phospho-AKT (Ser473) and phospho-glycogen synthase kinase 3 beta (GSK3β (Ser9)) did not show significant changes in liver of old p38 knock out old mice (Fig 5B). Thus, AKT activity is not affected by MK2 inactivation and hence, it seems that the AKT pathway is not related to the impairment of the actin cytoskeleton in these mice.

### Long-term p38α deficiency drives to Hsp27-dependent loss of actin polymerization

Heat shock protein 27 (HSP27) is another downstream target of p38α than can be also activated by MK2 and regulates the stability of actin filaments [58]. Phosphorylation of HSP27 was strongly diminished in old p38α knockout mice (Fig 5B), in which the most dramatic F-actin disassembly was found.

**Fig 6:**
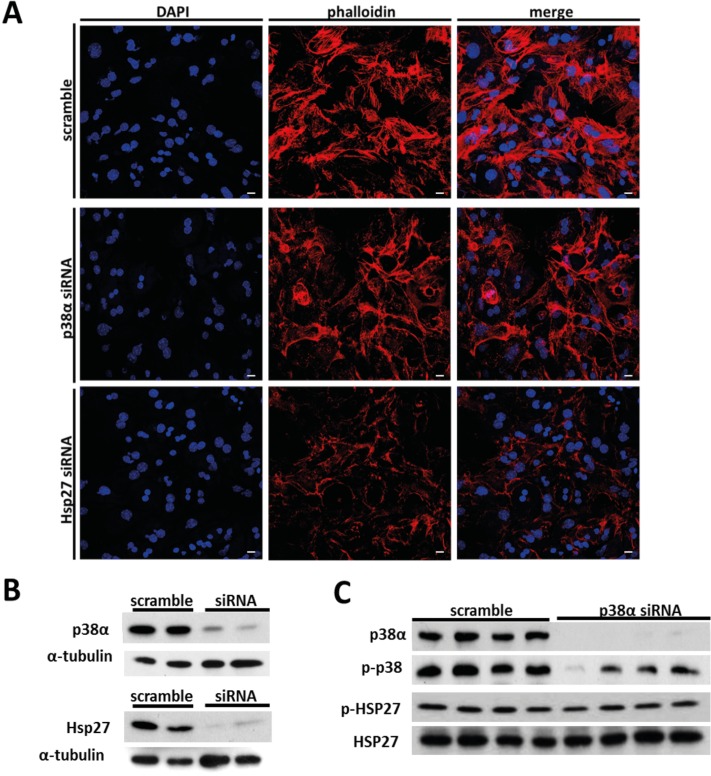
Actin polymerization in isolatedhepatocytes*p38α MAPK-*silenced and *Hsp27*-silenced. **a.** Representative image of actin filaments staining by phalloidin (red) and DAPI (blue) in isolated hepatocytes treated with scramble, *p38α MAPK* siRNA and *Hsp27* siRNA (Scale bars = 1000 μm). **b.** Silencing of *p38α MAPK* and *Hsp27 targets* by siRNA in isolated hepatocytes. **c.** Isolated hepatocytes scramble-treated and *p38α MAPK* siRNA-treated were Western blotted for p-p38 (Thr180/Tyr182), p38α, p-HSP27 (Ser82) and HSP27.

**Fig 7:**
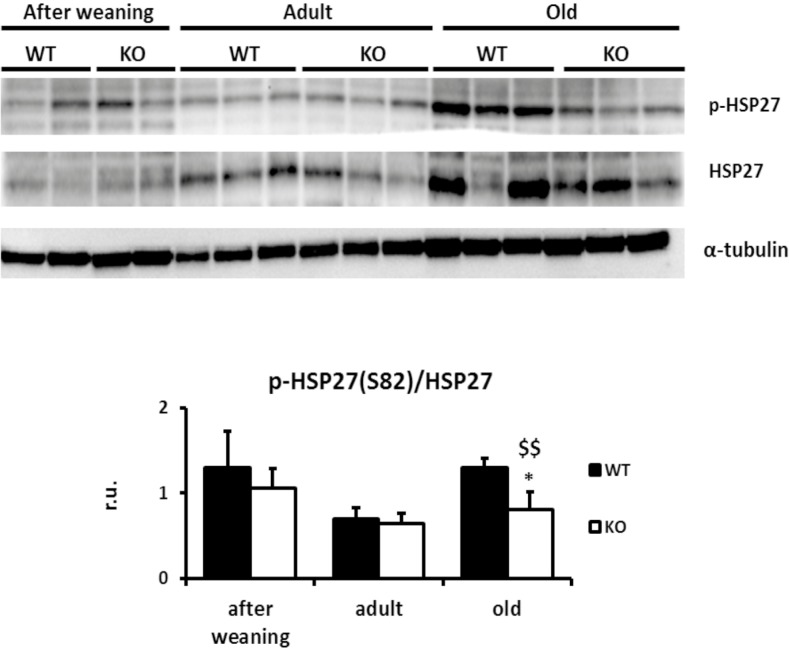
Only the *in vivo* animal model with a long-term p38α deficiency drives to inactivation of HSP27. Wild type and p38α knock out livers of all groups of age were Western blotted for p-HSP27 (Ser82) and total HSP27. α-tubulin was used as a loading control. Data are shown as mean ± SD. *P < 0.05 WT *versus* KO; $ $P < 0.01 old *versus* adult.

In order to confirm the role of p38α and HSP27 in actin polymerization, these targets were silenced by siRNA in primary cultures of hepatocytes (Fig 6B). *Hsp27* silencing decreases the number and length of actin filaments in hepatocytes whereas no changes were found when *p38α MAPK* was silenced (Fig 6A). Strikingly, phosphorylation levels of HSP27 remained unchanged in p38α-silenced hepatocytes (Fig 6C). Accordingly, we measured phosphorylation levels of HSP27 in the liver of WT and p38α knockout mice at all ages. Interestingly, the decrease in HSP27 phosphorylation was specific for the long-term p38α deficiency, as it was only found in old mice but not after weaning or adult mice (Fig 7).

## Discussion

Polyploidy accompanies late fetal development and postnatal maturation of hepatocytes [4, 5], reaches a plateau at maturity, and increases later on with aging [1]. This is a physiological feature of the liver and the increase in polyploidy that occurs with age is to a great extend independent of p38α. Nevertheless, in our model, in each group of age studied, the binucleation rate increases in p38α knock out mice compared with their corresponding wild type counterparts, especially upon aging. The lack of p38α leads to decreased liver mass in old mice without apparent changes in hepatocyte size. Moreover, the TUNEL staining and the cleaved PARP/PARP ratio remained unaffected upon p38α-deficiency in old mice showing that the reduced liver mass observed in our model should not be ascribed to apoptosis. Therefore, we hypothesized that inactivation of p38α should impair hepatocyte proliferation by affecting cell division in old mice.

It is known that p38α regulates the G_1_/S and G_2_/M cell cycle checkpoints prior to DNA synthesis and cell division, respectively [43–45, 59]. Accordingly, we have analyzed these checkpoints of the cell cycle. Livers from p38α knock out old mice showed up-regulation of cyclin B1, but they did not exhibit cyclin D1 overexpression. Cyclin D1 is a marker of the G_1_/S transition, whereas cyclin B1 is a marker for G_2_/M transition [59]. Hence, increased B1 levels suggest that hepatocytes from p38α knock out mice enter more actively into mitosis. In fact, increased proliferation have been considered hallmark of p38α-deficient cells [46] and mice with liver-specific deletion of p38α exhibited enhanced hepatocyte proliferation [47] and developed more liver tumors [48]. However, the highest binucleation rate observed in our model would suggest that although hepatocytes from p38α knock out mice enter actively into mitosis, fail to complete cell division, particularly cytokinesis. Interestingly, although incomplete cytokinesis is a common phenomenon in hepatocytes explaining the presence of hepatic polyploidy in the liver [1], under certain circumstances polyploidy is considered a protumoral feature that may give rise to cancer [60].

It is known that the increase in ploidy rate and cytokinesis failure that occurs after weaning was associated with activation of the AKT pathway [5]. Indeed, primary hepatocytes cultured with PI3K and AKT inhibitors reduced these failed cytokinetic events [61]. The activation of RhoA at the division site induced by AKT inhibition allowed proper cytoskeleton reorganization [62], and thus, successful cytokinetic performance was achieved [61]. In fact, it has been reported that although the PI3K-AKT pathway is required for G_2_/M progression, its inactivation is necessary for mitotic exit [63]. In addition, AKT downstream targets such as mammalian target of rapamycin complex 2 (mTORC2) and GSK3β have been described as cytoskeleton regulators [56, 57, 64]. However, the p38α-dependent hepatic ploidy should not be ascribed to stimulation of the AKT pathway because it remained unaffected. Hepatocyte binucleation triggered by p38α-deficiency at all ages could be ascribed to reduced activation of MNK1, which should affect the final step of cytokinesis, i.e. abscission of the intercellular bridge. Indeed, MNK1 is activated by phosphorylation directly triggered by p38 MAPK [65] and it is required for localization of centriolin to the midbody and subsequent abscission [55]. Accordingly, its silencing induced the formation of multinucleated cells due to cytokinesis failure [55]. Hence, our results suggest that MNK1 is a critical target of p38α whose inactivation would lead to binucleated cells at all ages, but especially upon aging.

During cytokinesis, actin dynamics play a critical role in animal cells [12]. At the end of mitosis, the actin network rearranges at the cleavage furrow and composes the contractile ring, which is essential in the process of cytokinesis [13]. Our results show that the long-term absence of p38α severely impairs actin cytoskeleton leading to a high presence of binucleated hepatocytes. The abnormalities in F-actin polymerization start in adult p38α knock out hepatocytes, and become severe in old p38α knock out mice. In fact, in these animals the G-actin/F-actin ratio was significantly increased. Thus, the severity of damage in actin cytoskeleton in p38α knock out old mice could explain the higher differences in the binucleation rate in comparison with wild type animals upon aging. In young animals, the effect of p38α deletion in actin cytoskeleton is more moderate and could not explain the differences observed in the ploidy in this group of age. The main mechanism that explains how the impairment of the actin cytoskeleton can drive to blockage of cytokinesis execution is related to the cleavage furrow formation [66]. The exact structure of the cleavage furrow remains unclear but several studies have shown how F-actin is required to interact with myosin and other scaffolding proteins such as septins and anillin during furrow formation [66, 67]. Moreover, cytokinesis completion depends on actin dynamics in the furrow as well as on the preexisting actin filaments that are nucleated outside the cleavage site [66]. Hence, when stimulation of actin filament assembly fails and actin dynamics is impaired, blockage of cytokinesis takes place.

**Fig 8:**
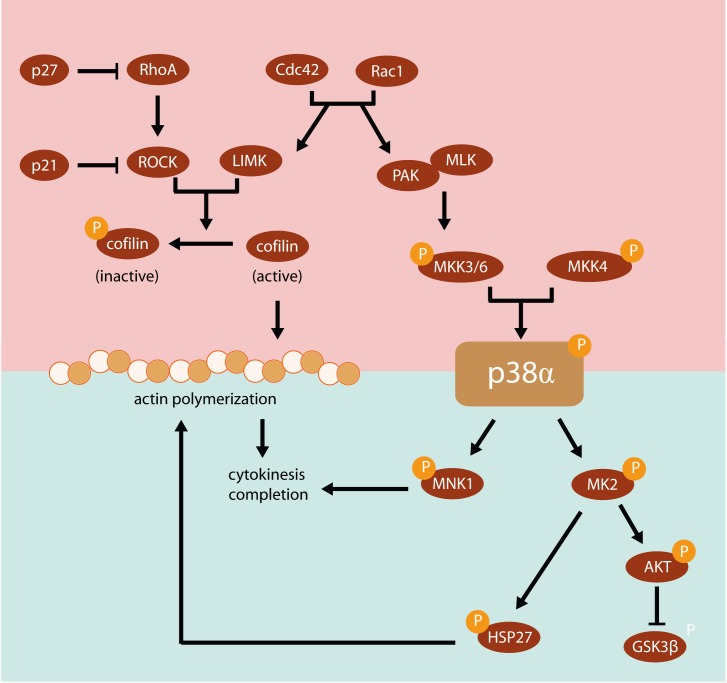
Scheme of the p38α-mediated phosphorylation pathways involved in the regulation of actin cytoskeleton. The Rho family plays a central role in organizing the actin cytoskeleton and in the regulation of cytokinesis. RhoA activity may be inhibited by p27, and additionally the RhoA downstream pathway may be blocked by p21 or cofilin. MNK1 and MK2 are major downstream targets of the p38α pathway that have been implicated in the regulation of cytokinesis and actin dynamics. HSP27 is another downstream target of p38α than can be also activated by MK2 and regulates the stability of actin filaments.

Rho family GTPases are essential regulators of actin dynamics during the cell cycle, especially during cytokinesis [13, 16] organizing the assembly of the contractile ring and triggering the actomyosin-driven constriction of the cleavage furrow [12, 18, 67]. In fact, significant deficiencies in F-actin polymerization could also be due to inactivation of the RhoA pathway [17]. Rac1 and Cdc42 GTPases may also regulate the assembly or disassembly of filamentous F-actin and importantly may activate p38 through p21-activated kinase 1 (PAK1) [13]. p38α MAPK also acts as a downstream target of the Rho family, and particularly of its three major members RhoA, Rac1, and Cdc42 *via* mixed lineage kinases (MLKs) [68] (Fig 8). Thus, p38 activation is likely to contribute to the biological effects of Rac and Cdc42 on actin cytoskeleton, affecting cell growth and proliferation, and regulating feedback loops [66].

Our findings show that long-term p38α deficiency triggers abnormal hyperactivation of both RhoA and Cdc42 GTPases, which is likely to be caused by p38α-dependent blockade of their downstream pathways. Indeed, RhoA activity triggers proteasome-mediated degradation of p21, which directly inhibits RhoA kinase (ROCK) [53] (Fig 8). However, p21 levels markedly increased upon long-term p38α deficiency, which should cause downstream down-regulation of the RhoA pathway. It has been reported that F-actin disruption stabilizes and enhances p21 levels [53] and this could explain the observed increase in p21 levels. In any case, the absence of net p21 degradation indicates that the RhoA pathway is blocked downstream at certain step because it is unable to trigger p21 degradation. On the other hand, hyperactivation of Cdc42 and dual specificity mitogen-activated protein kinase kinase 3/6 (MKK3/6) when p38α is absent would indicate the existence of a positive feedback loop that induces upstream the pathway Cdc42-PAK1-MKK3/6 that normally would lead to p38 activation [69] (Fig 8). Moreover, the hyperactivity of Cdc42 might interfere with cytokinesis since Cdc42 inhibition is considered necessary for cytokinesis completion [70, 71].

In regard to cofilin, increased phosphorylation in the nucleus would inhibit the actin-depolymerizing activity of nuclear cofilin in old p38α knock out mice [72], thus protecting F-actin polymerization in the nucleus where is implicated in several nucleus processes related with transcription and gene expression regulation specially during cell cycle [73–77] (Fig 8).

The dramatic loss of actin cytoskeleton in the cytosol associated with severely impaired actin polymerization upon aging could be ascribed to decreased phosphorylation of HSP27. HSP27 is a highly conserved oligomeric protein that has a critical function in the equilibrium between polymerization and depolymerization of actin filaments [58]. HSP27 displays actin-capping activity that is inhibited by phosphorylation [78], and thus phosphorylation of HSP27 markedly modifies this equilibrium in favor of polymerized actin, contributing to the maintenance of the microfilament network and to formation of the cleavage furrow [58] (Fig 8). In hepatocarcinoma cells, p38-mediated activation of HSP27 promotes migration and invasion because of the promotion of actin remodeling [79, 80]. Accordingly, activation of p38 MAPK in cells exposed to cytochalasin D increased the stability of the actin microfilaments in a HSP27 phosphorylation-dependent manner [58]. RhoA/Rho kinase pathway can promote p38α-mediated HSP27 phosphorylation in rabbit facial vein [81], and in in osteoblasts [81–83]. The association between HSP27 and RhoA was already described in muscle cells [84, 85] modulating actin-myosin interaction [86]. Nevertheless, in CCl39 cells p38 phosphorylation and HSP27 phosphorylation occurred independently of the Rho pathway [58]. Our results suggest that upon aging p38α induces HSP27 phosphorylation in order to keep the appropriate F-actin network for successful cytokinesis, whereas p38α deficiency enhances binucleation with age. MK2 may mediate p38α-dependent HSP27 phosphorylation [87–89] (Fig 8). MK2 activation depends on phosphorylation of the activation loop (Thr222) and the regulatory domain (Thr334), being the latter required for migration of the p38-MK2 complex from the nucleus to the cytoplasm [90]. As the lack of p38α in liver leads to absence of MK2 phosphorylation on Thr334 and to an age-dependent decrease of HSP27 phosphorylation, alteration of actin cytoskeleton would be expected. p-38 independent redundant mechanisms involved in HSP27 phosphorylation should maintain its normal levels in young and adult mice, but these redundant mechanisms would fail in the liver of old animals. Accordingly, silencing of Hsp27 in hepatocytes abrogated the actin microfilament network but neither Hsp27 phosphorylation nor actin cytoskeleton, were affected when p38α was silenced. This result could be explained because we have employed a transient p38 silencing method. In fact, only the *in vivo* animal model with a long-term p38α deficiency drives to inactivation of HSP27 thus disturbing cytoskeleton dynamics.

In conclusion, long-term p38α deficiency severely impairs actin cytoskeleton inducing actin disassembly and cytokinesis failure by reducing both HSP27 phosphorylation and MNK1 phosphorylation in the liver of old animals. Thus, p38α is essential to maintain in actin dynamics with age in hepatocytes. In addition, long-term p38α deficiency triggered RhoA and Cdc42 hyperactivation, but increased p21 levels that may inhibit downstream the RhoA pathway. The dramatic loss of actin cytoskeleton observed upon p38α deficiency with age should be taken into account when using p38 inhibitors for chronic therapies in the clinical practice.
